# Mitochondrial genome of the Boulenger's Slug-eating snake *Pareas boulengeri* (Serpentes: Pareidae)

**DOI:** 10.1080/23802359.2020.1804471

**Published:** 2020-08-12

**Authors:** Ruyi Huang, Lifang Peng, Diancheng Yang, Zhang Yong, Song Huang

**Affiliations:** a Shanghai Jian Qiao University, Shanghai, China; b Huangshan Noah Biodiversity Institute, Huangshan, China; c College of Life Sciences, Anhui Normal University, Wuhu, China

**Keywords:** Pareidae, *Pareas boulengeri*, mitogenome

## Abstract

The Boulenger's Slug-eating snake *Pareas boulengeri* is a species of snake in the family Pareidae. The complete mitochondrial genome sequence (mitogenome) of this species was determined by shotgun sequencing. The total length of mitogenome is 16,778 bp and contains 13 protein-coding genes, 22 tRNA genes, two ribosome RNA genes, and two control regions (CR). Its base composition was 31.6% for A, 24.6% for T, 14.5% for G and 29.3% for C. All the protein-coding genes in *P*. *boulengeri* were distributed on the H-strand, except for the ND6 subunit gene and eight tRNA genes which were encoded on the L-strand. The phylogenetic tree of *P*. *boulengeri* and 13 other related species was reconstructed using the maximum-likelihood (ML) method. The DNA data presented here will be useful to study the evolutionary relationships of *P*. *boulengeri*.

*Pareas* Wagler, 1830 is a genus of Asian snakes in the family Pareidae, which contains 15 species. All species in the genus *Pareas* are oviparous and harmless to humans (Wogan and Vogel 2012). They are nocturnal and mostly arboreal, and as the common name suggests, they feed exclusively on snails and slugs. None complete mitochondrial genome was reported about this genus. *Pareas boulengeri* (Angel 1920) was described as *Amblycephalus boulengeri* by Angel (1920) from Guizhou, China. It is relatively widespread in China (Gansu, Sichuan, Guizhou, Guangxi, Guangdong, Hunan, Henan, Jiangxi) (Zhao et al. 1998; Chen et al. 2006; Guo and Deng 2006; Zhao 2006). In this paper, we determined and described the mitogenome of *P*. *boulengeri* in order to obtain basic mitochondrial genetic information of this species.

The specimen of *P*. *boulengeri* was collected from Huangjialing Villege, Qimen County, Anhui Province, China (29°49′19″N, 117°32′24″E, 218 m a. s. l.). Fresh liver tissues were removed and immediately preserved in 95% ethanol. Total genomic DNA was extracted from muscle using a Qiagin DNEasy blood and tissue extraction kit (Qiagen Inc., Valencia, CA). The specimen and total genomic DNA were preserved and deposited in the Museum of Anhui Normal University (Voucher numbers: HSR18074). The total length of the complete mitogenome *P*. *boulengeri* (Genbank accession number MN866896) was sequenced to be 16,779 bp which consisted of 13 typical vertebrate protein-coding genes, 22 transfer RNA (tRNA) genes, two ribosomal RNA (rRNA) genes, and two D-loop, which is similar to the typical mtDNA of snakes and other vertebrates (Boore 1999; Sorenson et al. 1999). The overall base composition of the entire genome was as follows: A (31.6%), T (24.6%), C (29.3%) and G (14.5%). One of the 13 PCGs (nad6) and eight tRNAs (trnA, trnC, trnE, trnN, trnP, trnQ, trnS2, and trnY) were encoded on the L-strand, whereas the other 29 genes, including 12 PCGs, 14 tRNAs, two rRNAs, and two D-loop, were encoded on the H-strand. The positions of RNA genes were predicted by the MITOS (Bernt et al. 2013), and the locations of protein-coding genes were identified by comparing with the homologous genes of other related species.

To further validate the new determined sequences, we selected the 12 protein-coding genes located on heavy strand except for ND6 which encoded on the light strand of *P*. *boulengeri* in this study, and together with other 13 related species from GenBank to perform phylogenetic analysis. These species were as follows: *Dinodon rufozonatum*, *D*. *semicarinatus*, Lycodon ruhstrati, *L*. *flavozonatus*, *Ptyas dhumnades*, *Cyclophiops major*, *Euprepiophis perlacea*, *Elaphe poryphyracea*, *Oocatochus rufodorsatus, Pituophis catenifer*, *E*. *schrenckii*, *E*. *anomala*. we used *E*. *schrenckii* and *E*. *anomala* as an outgroup. We aligned the new sequences of *P*. *boulengeri*, as well as other sequences retrieved from GenBank using MEGA X (Kumar et al. 2018). Maximum-likelihood (ML) method was used to reconstruct phylogenetic tree (Figure 1) in http://www.phylo.org/portal2/login!input.action. The phylogenetic analysis result was consistent with the previous research with a high support. It indicated that our new determined mitogenome sequences could meet the demands and explain some evolution issues.

**Figure 1.**
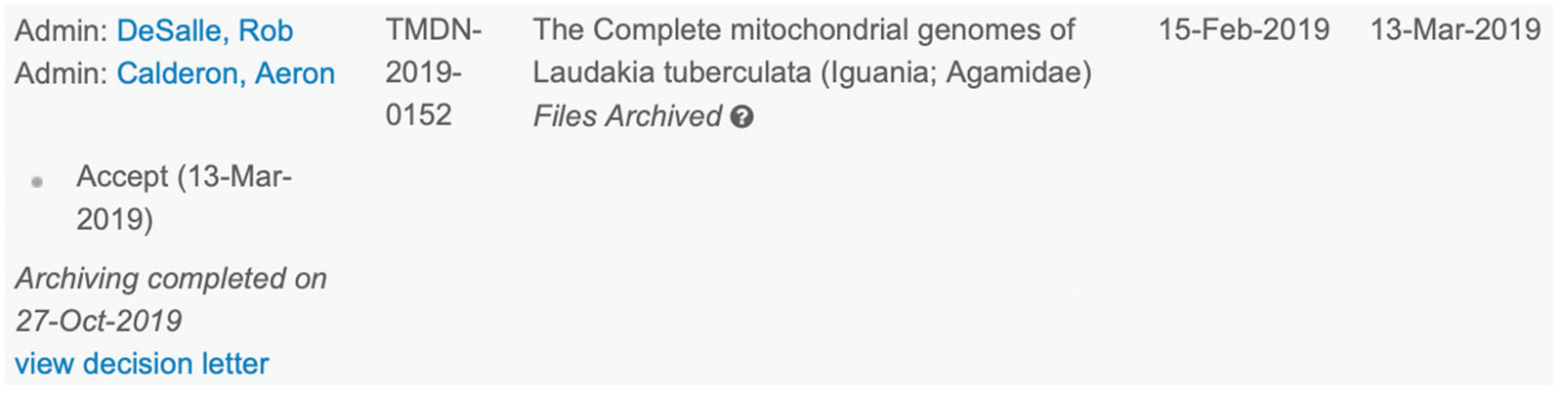
A maximum likelihood (ML) tree of Pareas boulengeri in this study and other related species was constructed based on the dataset of the 12 protein-coding genes by online tool RAxML. The numbers above the branch meant bootstrap value. Bold black branches highlighted the study species and corresponding phylogenetic classification. The analyzed species and corresponding NCBI accession number as follows: Dinodon rufozonatum (KJ179950), D. semicarinatus (AB008539), Lycodon ruhstrati (MK867843, KJ179951), L. flavozonatus(KR911720), Ptyas dhumnades (KF148620), Cyclophiops major (KF148620), Euprepiophis perlacea (KF750656), Elaphe poryphyracea (GQ181130), Oocatochus rufodorsatus (KC990020), Pituophis catenifer (KU833245), E. schrenckii (KP888955), E. anomala (KP900218).

## Data Availability

The data that support the findings of this study are openly available in GenBank of NCBI at https://www.ncbi.nlm.nih.gov, reference number MN866896.

## References

[CIT0001] Angel MF. 1920. Liste de reptiles récémment déterminés et entrés dans les collections et description d’une nouvelle espèce du genre Amblycephalus. Bull Mus nation. Hist nat. Paris 1920:112–114.

[CIT0002] Bernt M, Donath A, Juhling F, Externbrink F, Florentz C, Fritzsch G, Putz J, Middendorf M, Stadler PF. 2013. MITOS: improved de novo metazoan mitochondrial genome annotation. Mol Phylogenet Evol. 69(2):313–319. 22982435 10.1016/j.ympev.2012.08.023

[CIT0003] Boore JL. 1999. Animal mitochondrial genomes. Nucleic Acids Res. 27(8):1767–1780. 10101183 10.1093/nar/27.8.1767 PMC148383

[CIT0004] Chen XH, Zhu MW, Hou MG, Qu WY. 2006. A new record of Colubridae family, *Pareas boulengeri*, in Henan Province. Sichuan J Zool. 25(2):269. (In Chinese)

[CIT0005] Guo KJ, Deng XJ. 2006. A new record of Colubridae family, *Pareas boulengeri*, in Hunan Province. Sichuan J Zool. 25 (2):270. (In Chinese)

[CIT0006] Kumar S, Stecher G, Li M, Knyaz C, Tamura K. 2018. MEGA X: molecular evolutionary genetics analysis across computing platforms. Mol Biol Evol. 35(6):1547–1549. 29722887 10.1093/molbev/msy096 PMC5967553

[CIT0007] Sorenson MD, Ast JC, Dimcheff DE, Yuri T, Mindell DP. 1999. Primers for a PCR-based approach to mitochondrial genome sequencing in birds and other vertebrates. Mol Phylogenet Evol. 12(2):105–114. 10381314 10.1006/mpev.1998.0602

[CIT0008] Wogan G, Vogel G. 2012. The IUCN Red List of Threatened Species. Gland: IUCN.

[CIT0009] Zhao EM. 2006. Snakes of China. Hefei (China): Anhui Sciences and Technology Publishing House, p. 243–244. (In Chinese)

[CIT0010] Zhao EM, Huang MH, Zong Y, et al. 1998. Fauna Sinica (Reptilia vol. 3 Squamata: Serpentes) Beijing: Science Press. (In Chinese)

